# Supplementary material for: Utility and Mechanism of SHetA2 and Paclitaxel for Treatment of Endometrial Cancer
Source: Cancers (Basel). 2021 May 12;13(10):2322. doi: 10.3390/cancers13102322 (PMC8150795; doi:10.3390/cancers13102322)
Supplement: Supplementary file 1 [file cancers-13-02322-s001.zip › cancers-1189258 suppl .pdf]

cancers

Supplementary Files

Utility and Mechanism of SHetA2 and Paclitaxel for Treatment of Endometrial Cancer

Vishal Chandra, Rajani Rai and Doris Mangiaracina Benbrook

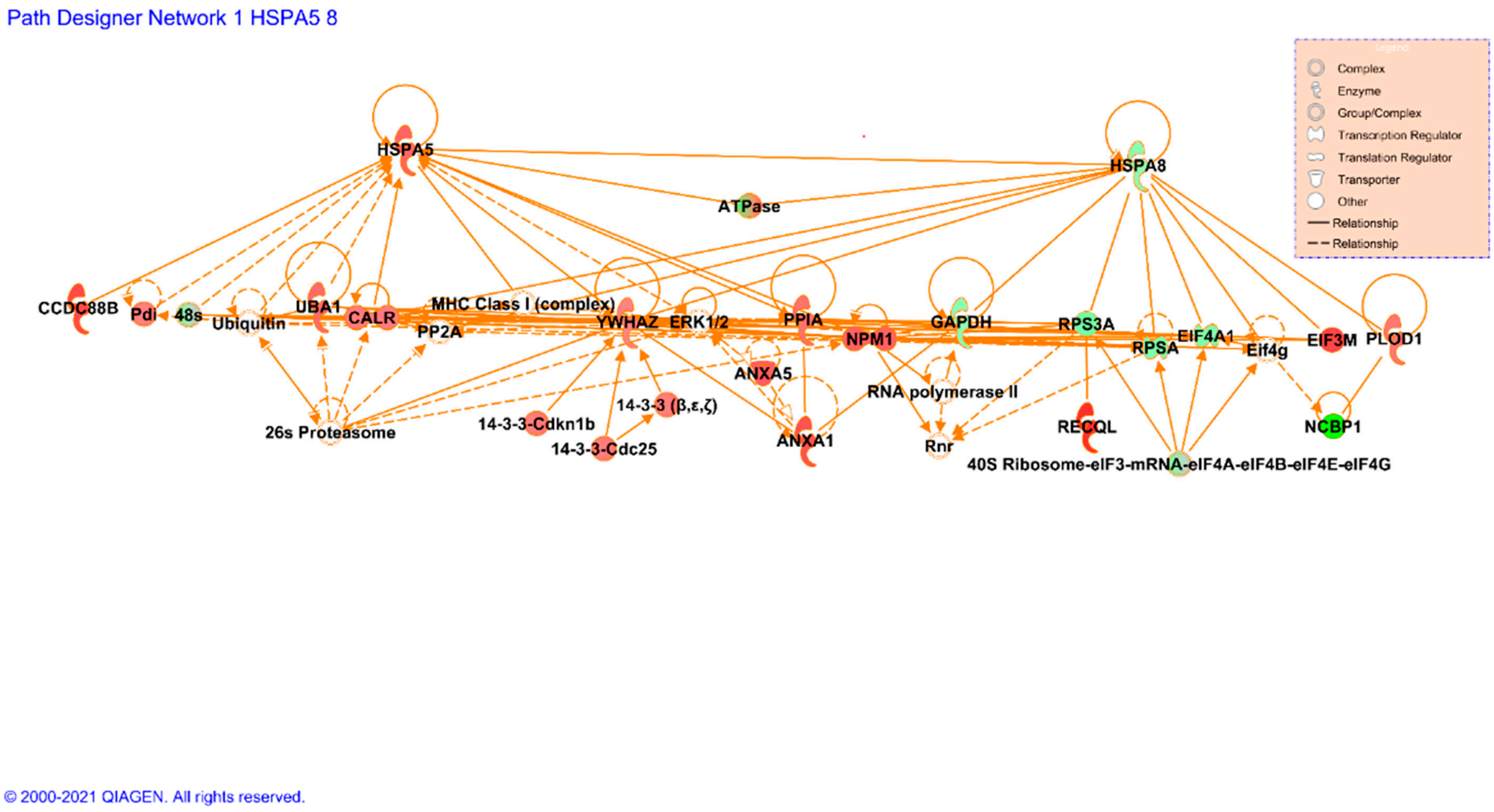

Figure S1. Network Interactions of Grp78 and hsc70 in SHetA2 proteomic effects in Ishikawa endometrial cancer cells.

Cell cycle Markers

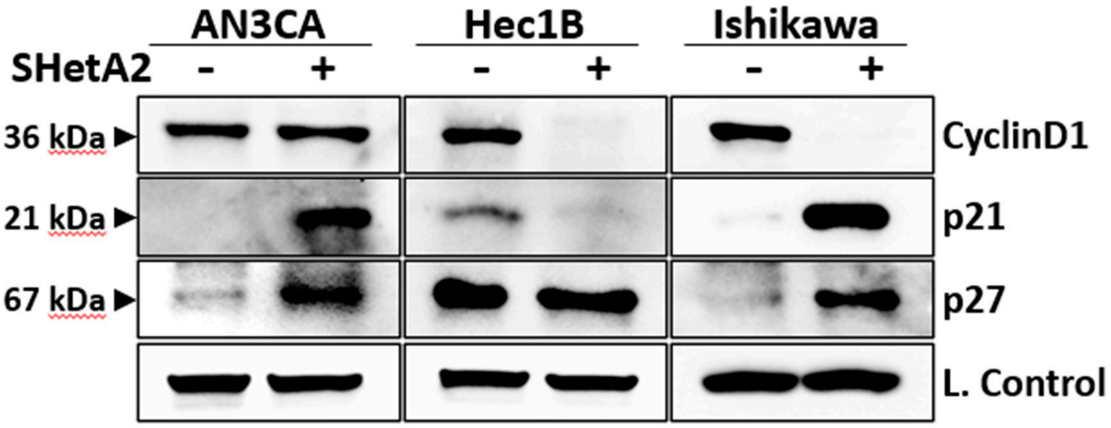

Loading Controls

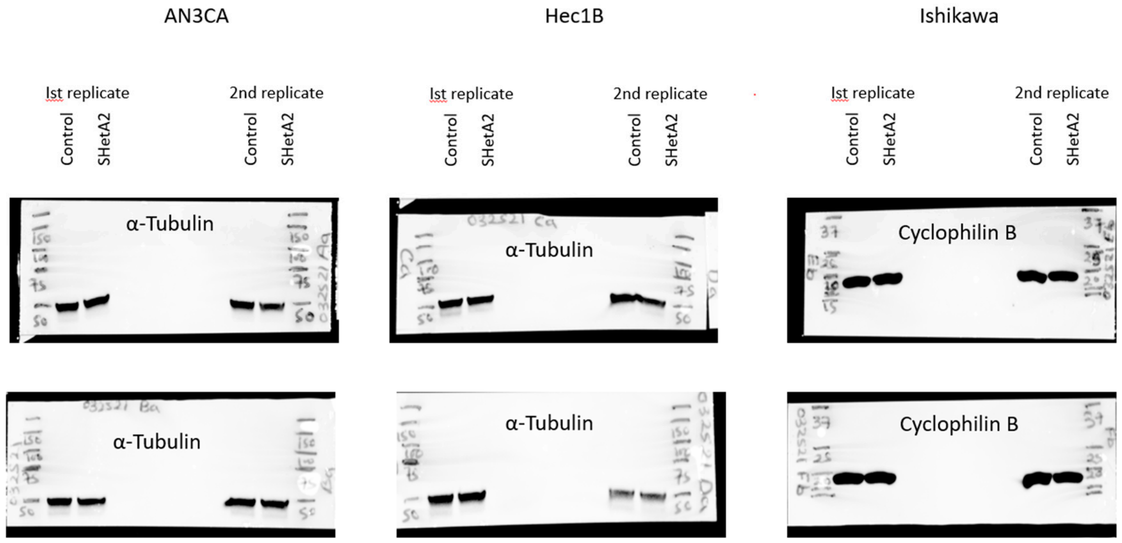

Cyclin D1

AN3CA

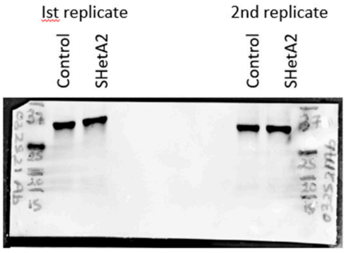

Hec1B

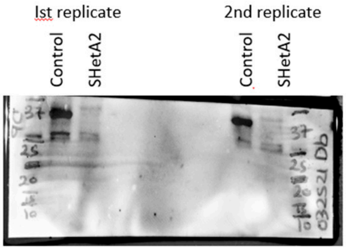

Ishikawa

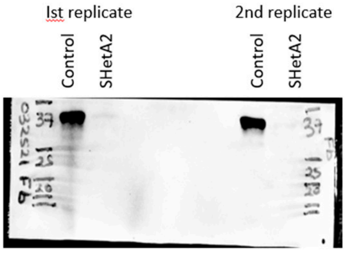

P21

AN3CA

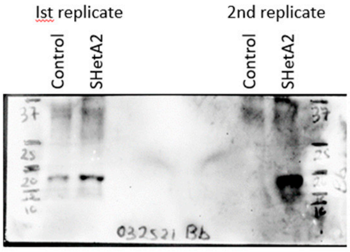

Hec1B

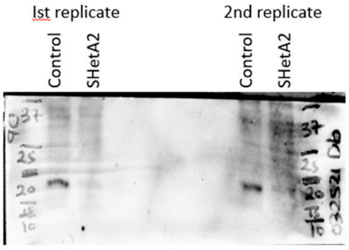

Ishikawa

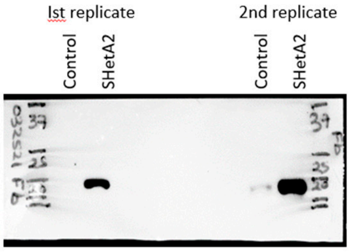

P27

AN3CA

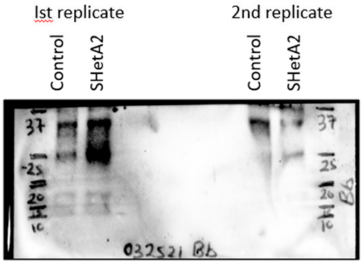

Hec1B

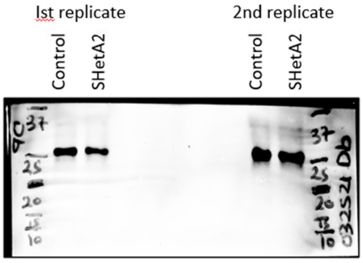

Ishikawa

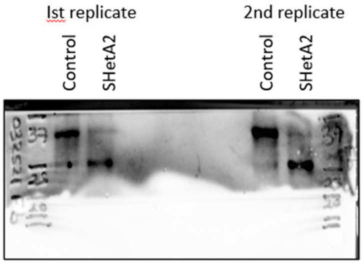

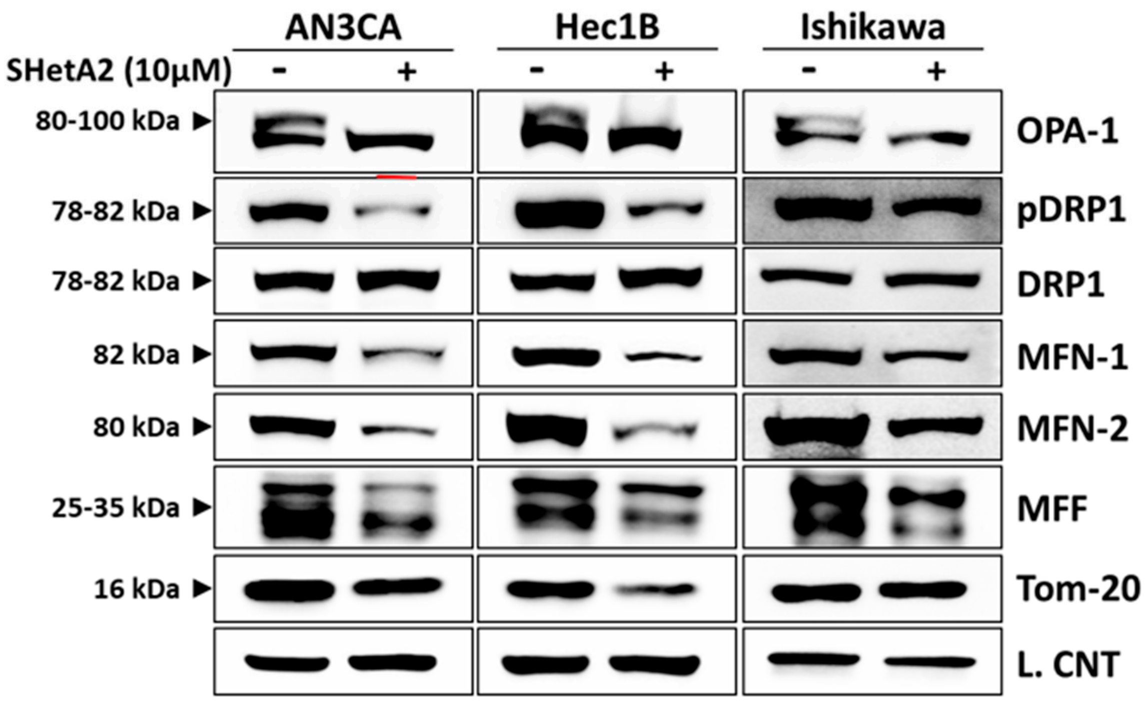

Loading Controls

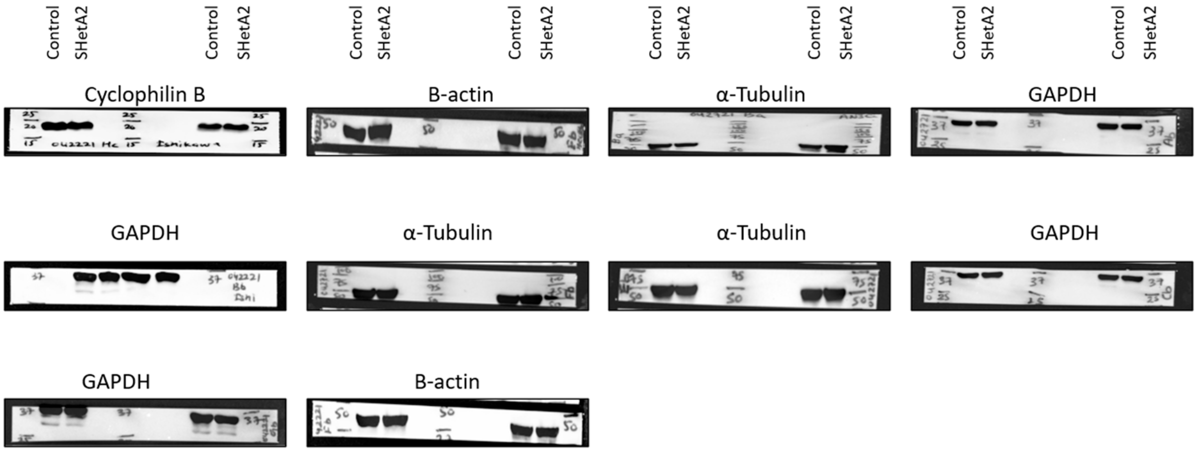

OPA1

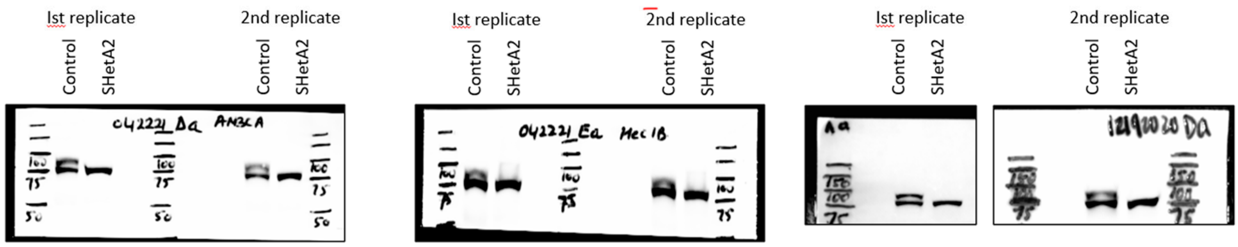

pDRP-1

AN3CA

Ist replicate — Control, SHetA2

2nd replicate — Control, SHetA2

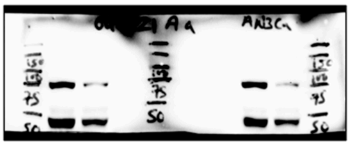

Hec1B

Ist replicate — Control, SHetA2

2nd replicate — Control, SHetA2

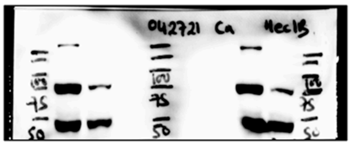

Ishikawa

Ist replicate — Control, SHetA2

2nd replicate — Control, SHetA2

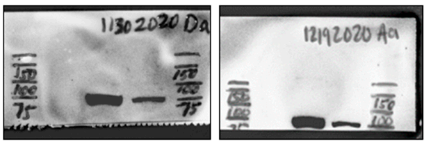

DRP-1

AN3CA

Ist replicate — Control, SHetA2

2nd replicate — Control, SHetA2

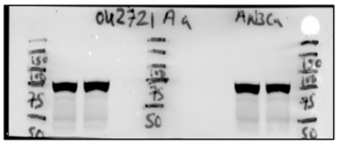

Hec1B

Ist replicate — Control, SHetA2

2nd replicate — Control, SHetA2

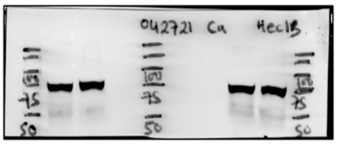

Ishikawa

Ist replicate — Control, SHetA2

2nd replicate — Control, SHetA2

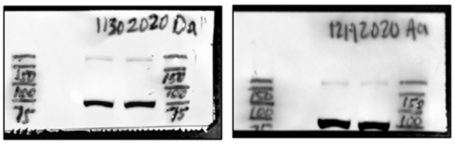

MFN-1

AN3CA

Ist replicate — Control, SHetA2

2nd replicate — Control, SHetA2

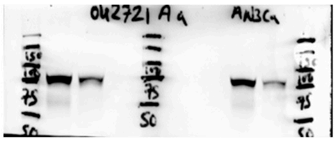

Hec1B

Ist replicate — Control, SHetA2

2nd replicate — Control, SHetA2

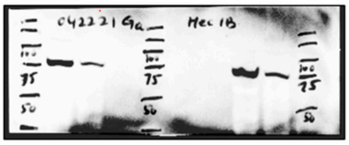

Ishikawa

Ist replicate — Control, SHetA2

2nd replicate — Control, SHetA2

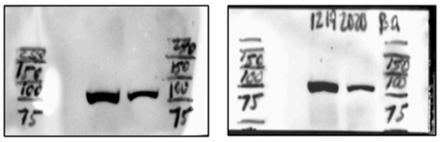

MFN-2

AN3CA

Ist replicate — Control, SHetA2

2nd replicate — Control, SHetA2

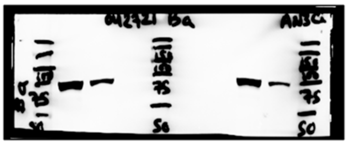

Hec1B

Ist replicate — Control, SHetA2

2nd replicate — Control, SHetA2

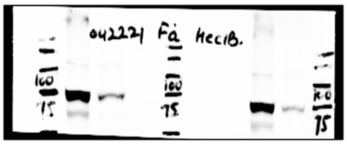

Ishikawa

Ist replicate — Control, SHetA2

2nd replicate — Control, SHetA2

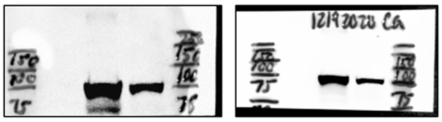

MFF

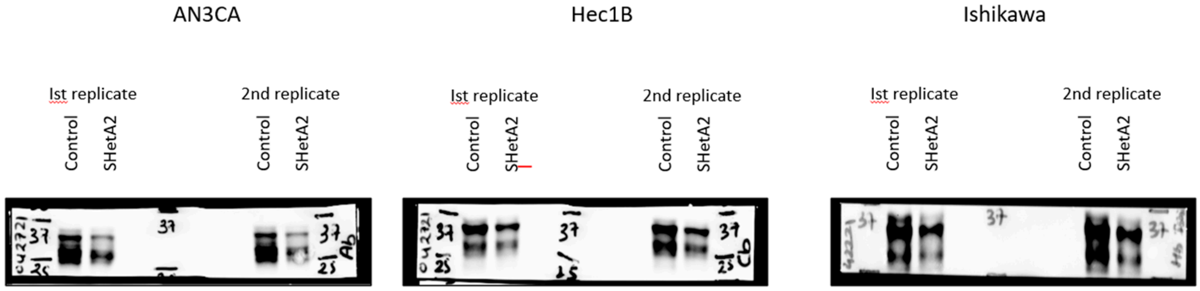
AN3CA
Hec1B
Ishikawa
Ist replicate
2nd replicate
Control
SHetA2

Tom20

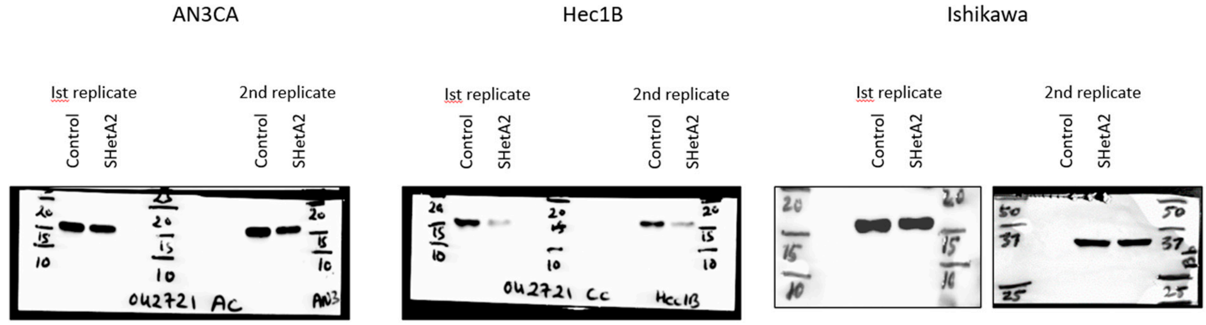
AN3CA
Hec1B
Ishikawa
Ist replicate
2nd replicate
Control
SHetA2

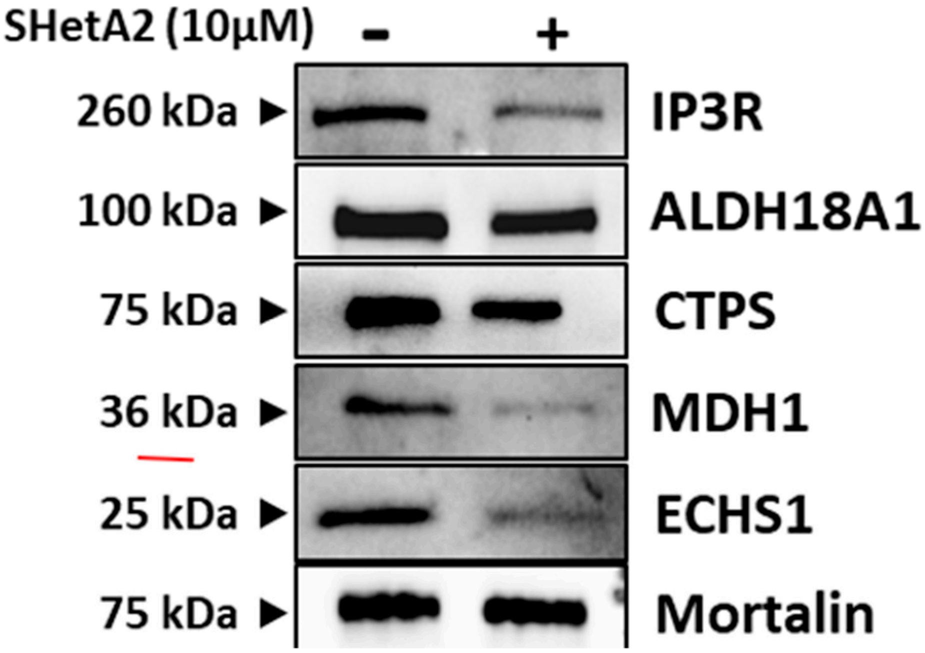
SHetA2 (10µM)
-
+
260 kDa
IP3R
100 kDa
ALDH18A1
75 kDa
CTPS
36 kDa
MDH1
25 kDa
ECHS1
75 kDa
Mortalin

Ishikawa
Co-IP results

IP3R

Ist replicate

Control SHetA2

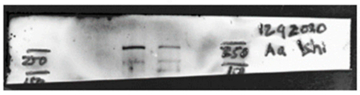

2nd replicate

Control SHetA2

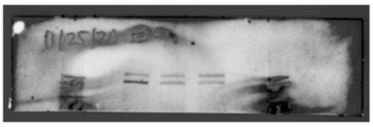

ALDH18A1

Ist replicate

Control SHetA2

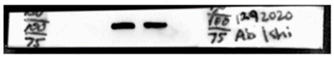

2nd replicate

Control SHetA2

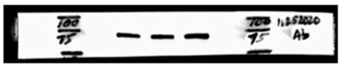

CTPS

Ist replicate

Control SHetA2

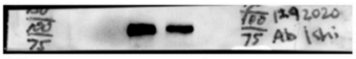

2nd replicate

Control SHetA2

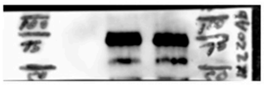

Ishikawa
Co-IP results

MDH1

Ist replicate

Control SHetA2

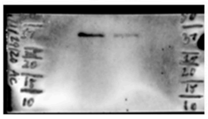

2nd replicate

Control SHetA2

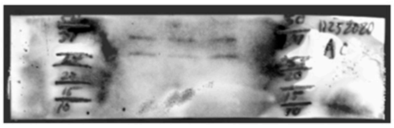

ECHS1

Ist replicate

Control SHetA2

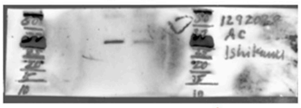

2nd replicate

Control SHetA2

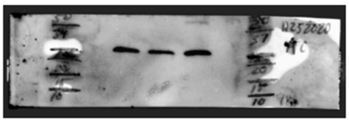

GRP75

Ist replicate

Control SHetA2

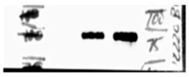

2nd replicate

Control SHetA2

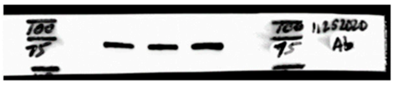

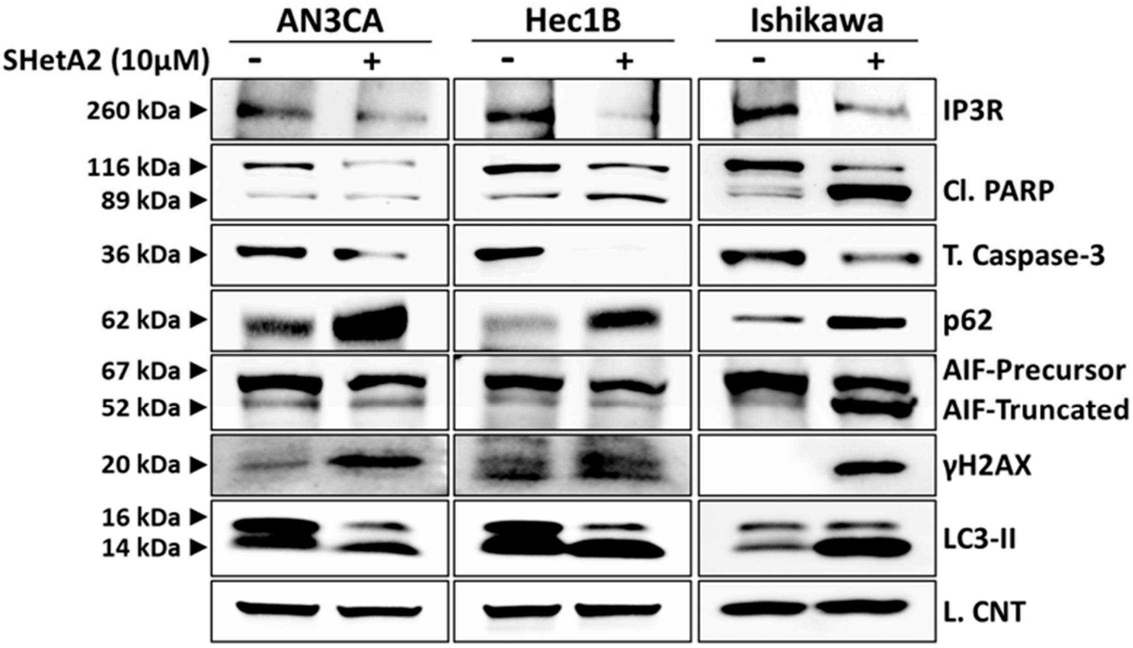

IP3R

AN3CA | Hec1B | Ishikawa

Ist replicate: Control, SHetA2 — 2nd replicate: Control, SHetA2 (for each cell line)

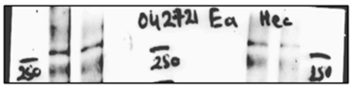

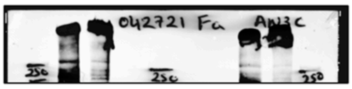

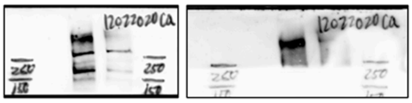

Total PARP

AN3CA | Hec1B | Ishikawa

Ist replicate: Control, SHetA2 — 2nd replicate: Control, SHetA2 (for each cell line)

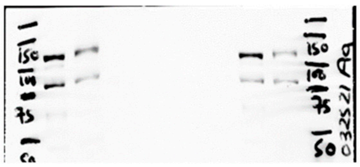

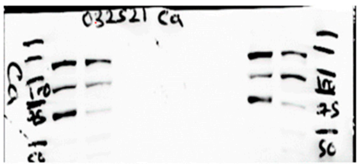

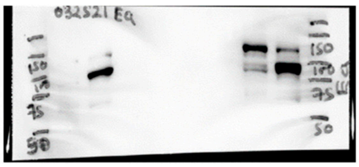

Total Caspase-3

AN3CA | Hec1B | Ishikawa

Ist replicate: Control, SHetA2 | 2nd replicate: Control, SHetA2 (for each cell line)

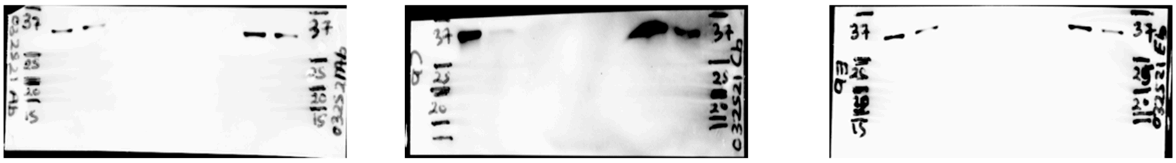

p62

AN3CA | Hec1B | Ishikawa

Ist replicate: Control, SHetA2 | 2nd replicate: Control, SHetA2 (for each cell line)

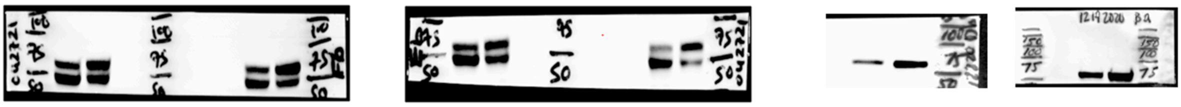

AIF

AN3CA | Hec1B | Ishikawa

Ist replicate: Control, SHetA2 | 2nd replicate: Control, SHetA2 (for each cell line)

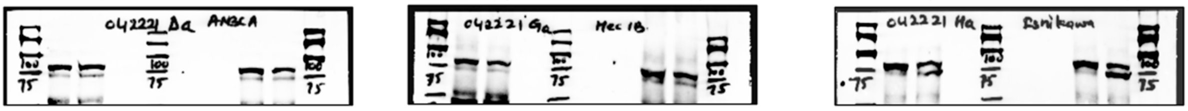

H2ax

AN3CA | Hec1B | Ishikawa

Ist replicate: Control, SHetA2 | 2nd replicate: Control, SHetA2 (for each cell line)

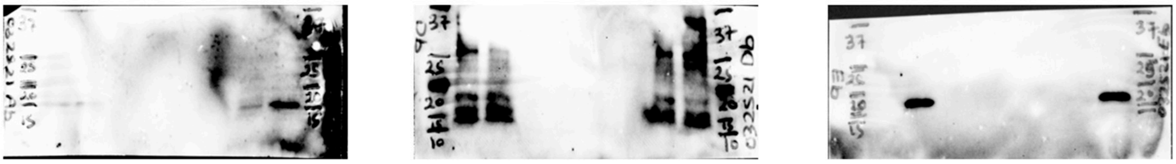

LC IIIB

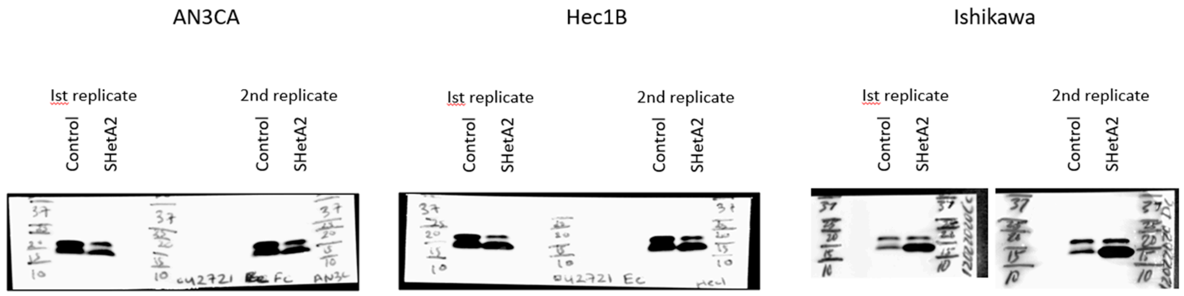

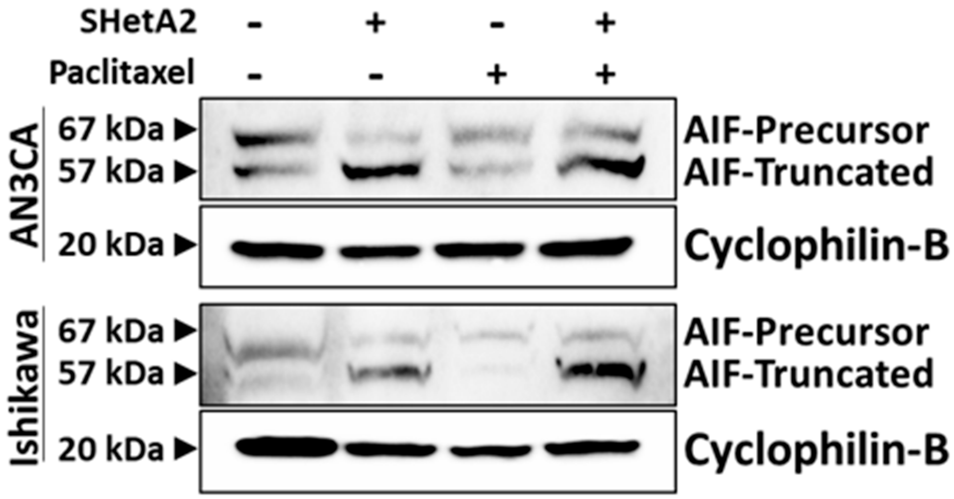

Loading Control

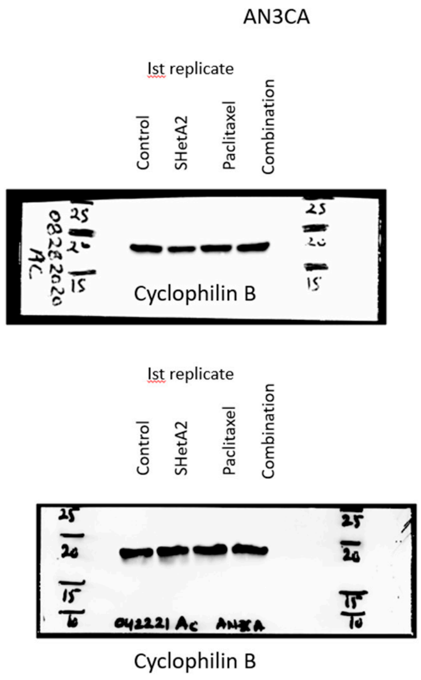

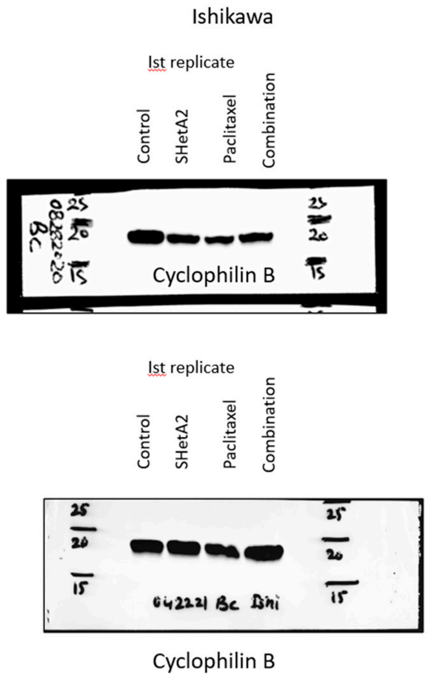

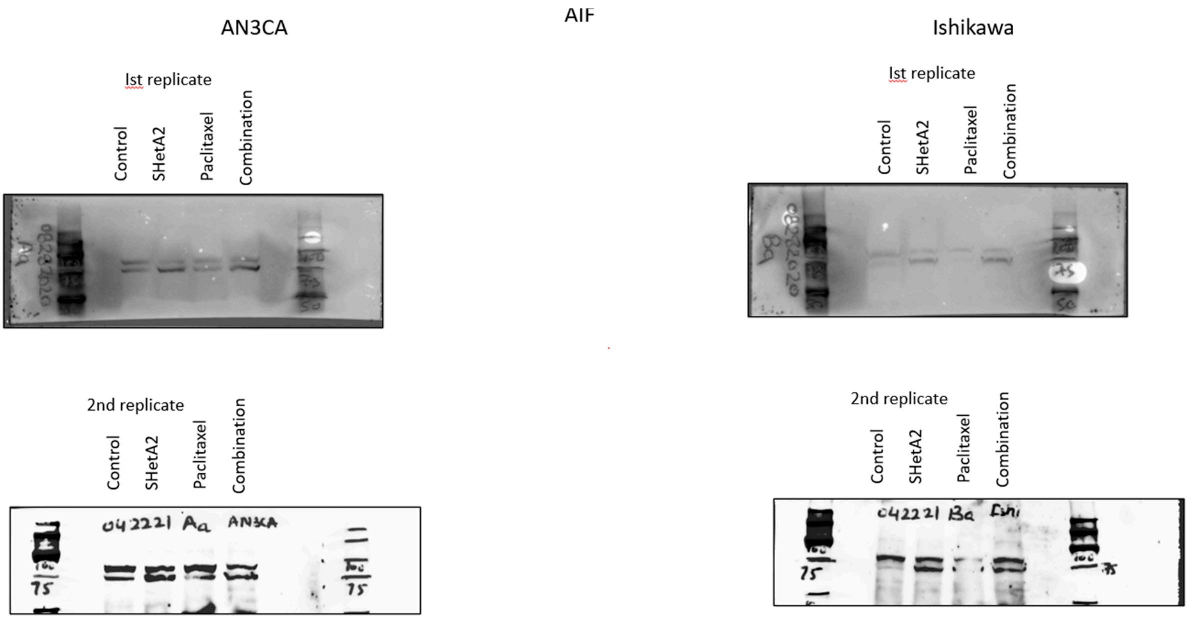

Figure S2. Full images of all Western blots of conducted in this study.
